# A preliminary study to evaluate efficacy and safety of Lugol’s solution following radioiodine for remnant ablation in differentiated thyroid cancer

**DOI:** 10.3389/fonc.2026.1783763

**Published:** 2026-02-25

**Authors:** Jiangfeng Wang, Yunyun Zhu, Quanyong Luo, Chentian Shen

**Affiliations:** Department of Nuclear Medicine, Shanghai Sixth People’s Hospital Affiliated to Shanghai Jiao Tong University School of Medicine, Shanghai, China

**Keywords:** differentiated thyroid cancer, efficacy, Lugol’s solution, radioiodine remnant ablation, safety

## Abstract

**Purpose:**

This randomized controlled trial aimed to determine if Lugol’s solution following radioactive iodine (RAI) therapy enhances the efficacy of ablation and mitigates radiation-induced toxicity in patients with differentiated thyroid cancer.

**Methods:**

In this prospective study, 97 patients were enrolled and randomized to control group (RAI) and test group (RAI + Lugol’s solution). The primary endpoint was the rate of successful ablation. The secondary endpoint was short-term (d3 and d10) and long-term (6- to 9-month after RAI therapy) adverse events (AEs).

**Results:**

The rate of negative DxWBS was similar between control and test group (93.3% vs. 91.2%, *p* = 0.748). At the 6- to 9-month follow-up, while the successful ablation rate (sTg <1 ng/mL) was numerically higher in the test group compared to the controls (82.8% vs. 66.7%, *p=*0.131), applying a stricter stimulated thyroglobulin (sTg) cutoff (<0.2 ng/mL) revealed a statistically significant advantage for the test group (65.7% vs. 40.0%, *p* = 0.041). Uni- and multi-variate analysis showed Lugol’s Solution administration significantly correlated with lower level of sTg at the 6- to 9-month follow-up. The two groups exhibited comparable short-term AEs rates (46% vs. 34%, *p* = 0.230) and profiles. The most common AEs included neck swelling, pain, loss of appetite and dry mouth. However, the control group reported 2 cases of long-term AEs, whereas none were observed in the test cohort.

**Conclusion:**

In this preliminary study, the addition of Lugol’s Solution following RAI showed non-inferior ablation efficacy with a numerically lower incidence of long-term AEs, despite numerically higher short-term AE rates.

**Clinical trial information:**

It was registered at Chinese Clinical Trial Registry with identifier ChiCTR1900027705 at November 24^th^, 2019.

## Introduction

Thyroid cancer is the most commonly diagnosed cancer in adolescents and in young female adults under 30-year-old, both worldwide and in China (1–3). Differentiated thyroid cancer (DTC) accounts for most cases of thyroid cancer (4). Over the past 30 years, the incidence of this tumor has been increased by 6-fold, which is mainly attributed to early diagnostic scrutiny (5, 6).

Radioactive iodine (RAI) therapy is selectively administered after thyroidectomy, based on a risk-adapted approach, to ablate the thyroid remnant and treat residual or metastatic disease (2, 7, 8). According to 2025 American Thyroid Association (2025 ATA) guideline, its use is routinely recommended for high-risk patients, while for those with intermediate or low risk, the decision is individualized, weighing the potential benefits against risks and patient preferences. Serum thyroglobulin is inherently a highly sensitive marker for differentiated thyroid tissue (residual or recurrent) and has good specificity. RAI ablation primarily improves the interpretability of thyroglobulin (Tg) measurements by reducing or eliminating remnant thyroid tissue, thereby enhancing its sensitivity and specificity as a marker of persistent or recurrent disease (9–11). However, it may result in a wide range of adverse events (AEs) at the same time. The main AEs are salivary and lacrimal glands dysfunction due to higher radioiodine uptake in these organs (12, 13). In addition, decreased thrombocytes and leucocytes or second primary malignancy may also be triggered (14).These potential toxicities pose a great threat to life quality, especially for younger patients with long life expectancy (15).

To optimize the clinical risk-benefit balance, numerous studies previously focused on dose optimization of radioiodine (^131^I) by comparing efficacy/safety between low and high activities of radioiodine (^131^I) (16, 17). In contrast, few studies are conducted to explore new approaches to enhance radioiodine accumulation in remnant thyroid, while decreasing accumulation in normal organs, such as other glands, bone marrow, etc.

In thyroid, acute excessive iodine intake reduces the organification of iodine and thyroid hormone secretion in thyroid, which is called “Wolff-Chaikoff effect” (18, 19). However, this rule did not apply to other tissues. Based on this, there is a hypothesis that the acute excessive stable iodine (Lugol’s Solution) after the RAI treatment may inhibit ^131^I release from remnant thyroid and boost its clearance from other tissues, such as salivary glands and bone marrow. Further, it may enhance efficacy and reduce toxicities of RAI therapy to the maximum extent.

As a result, this randomized controlled trial was conducted to compare the rate of successful ablation and side effects between “RAI” group and “RAI+ Lugol’s Solution” group, aiming to explore an innovative RAI practice pattern.

## Method

This single-center prospective study followed the principles in the Declaration of Helsinki and was approved by the Institutional Review Board of Ethics Committee of Shanghai Sixth People’s Hospital (Approval No.2019-142). It is registered at Chinese Clinical Trial Registry with identifier ChiCTR1900027705. Written consent has been obtained from each patient or subject after full explanation of the purpose and nature of all procedures used.

### Patients

Patients with DTC from our center were prospectively and consecutively enrolled from June 2020 to October 2023. The inclusion criteria were (1) patients aged 18-70, confirmed with DTC after thyroidectomy; (2) patients who received near-total or total thyroidectomy within 1–6 months; (3) patients with 2015 ATA low-risk or intermediate-risk thyroid cancer with lower risk features (i.e., with negative surgical margin, no other known gross residual disease or any other adverse features); (4) patients who plan to perform RAI therapy in our center;(5) patients with a stimulated thyroid stimulating hormone (TSH) level of ≥30 IU/mL following levothyroxine withdrawal for a minimum of 3 weeks.; (6) a stimulated thyroglobulin (sTg) level of 0–10 ng/mL (under the same stimulation conditions mentioned above), with a thyroglobulin antibody (TgAb) level ≤115 IU/mL; (7) patients who had low-iodine diet (LID) in preparation for RAI therapy for 2 weeks.

The exclusion criteria were (1) patients with aggressive tumor histology (e.g., tall cell, hobnail variant, columnar cell carcinoma); (2) tumor invasion of extrathyroidal tissues observed macroscopically during surgery; (3) presence of regional recurrence or distant metastases; (4) received enhanced CT scan with Iodinated contrast agents within 3 months; (5) patients who underwent RAI therapy before; (6) patients who received any treatment except operation and thyroid hormone replacement therapy, like radiotherapy; and (7) patients with a history of other malignancies.

### Study design

**Figure 1:**
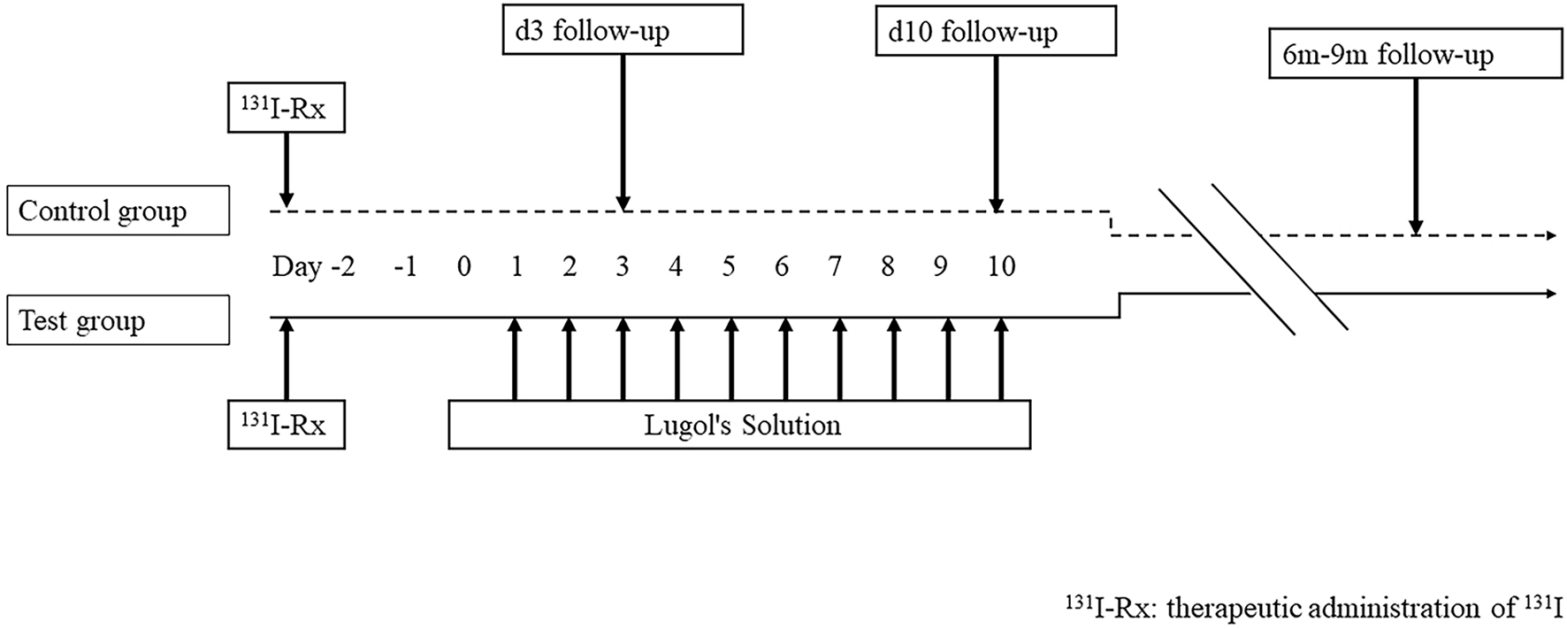
Study time line. Patients with previously thyroidectomy were consecutively enrolled and randomized to control group (RAI) and test group (RAI+ Lugol’s solution). All patients were administrated of 30 mCi ^131^I. After 72h, patients in the test group were administrated Lugol’s solution for 10 consecutive days (d1-d10). On d3, d10 and at 6- to 9- month follow-up, the adverse events (AEs) were recorded and assessed. At 6- to 9- month follow-up, diagnostic ^131^I whole-body scan (DxWBS) was performed and stimulated thyroglobulin (sTg) level were also measured. ^131^I-Rx, therapeutic administration of ^131^I.

The patients were randomized into two groups. The randomization sequence was generated by an independent statistician using a computer-generated random number list (SPSS, version 25.0, IBM Corp.), and allocation concealment was ensured using sequentially numbered, opaque, sealed envelopes. Patients in test group received ^131^I activity of 30 mCi and then had Lugol’s Solution (iodine 0.499 mol/L) 250uL 72 hours after ^131^I treatment, three times a day for ten days (d1-d10, d1 was defined as the first dosing day of compound iodine oral solution). Adherence to this regimen was monitored under supervision. Study nurses conducted daily telephone calls to confirm each dose was taken. Patients in control group received ^131^I activity of 30 mCi. All patients in this study resumed levothyroxine 48h after oral radioiodine therapy (^131^I). At the 6- to 9-month follow-up, radioiodine diagnostic whole-body scans (DxWBS), Single photon emission computed tomography with computed tomography (SPECT/CT) if necessary, was performed and sTg was measured. (**Figure 1**). AEs were recorded and assessed on d3, d10 and at 6–9 months after RAI therapy.

### Study endpoints

Primary endpoint of this study was successful ablation which was defined using structural and/or biochemical evidence at the 6- to 9-month follow-up. The following criteria were applied separately under adequate TSH stimulation (achieved by levothyroxine withdrawal for ≥3 weeks, resulting in TSH ≥30 IU/mL): (1) negative thyroid bed uptake on DxWBS, (2) sTg level <1 ng/mL, (3) sTg level <0.2 ng/mL, and (4) the combination of negative DxWBS with sTg <1 ng/mL (20).

Secondary endpoint were AEs recorded at d3, d10 (short-term AEs) and at the 6- to 9-month follow-up (long-term AEs). Trained investigators graded AEs using Common Terminology Criteria for Adverse Events (CTCAE) v5.0 guidelines.

### Statistical analyses

Continuous variables were expressed as the mean ± standard deviation (SD), and categorical variables were expressed as frequencies and percentages. Groups were compared for categoric data or frequency of events using the Pearson’s Chi-squared Test and for continuous variables using the Student’s *t* test. Linear regression analyses were performed for uni-variate and multi-variate analysis. All tests were 2-sided, and values of *p* less than 0.05 were considered statistically significant.

## Result

### Demographics and baseline characteristics

Totally, 97 patients were recruited in this research with 71 females and 26 males. As per 2025 ATA guideline, 91.8% (89/97) patient was classified into the low-intermediate and intermediate-high risk groups. There was no significant difference in the distribution of these risk categories between the control and test groups (*p* = 0.383) (**Table 1**). All demographics and baseline characteristics were well balanced between two groups, including age, sex, cancer histology, pathological T staging, N staging, and TNM staging, TSH and sTg prior to RAI. All patients were evaluated by cervical ultrasound prior to RAI administration, with no residual thyroid tissue identified in the thyroid bed.

**Table 1:** Patient and tumor characteristics.

Characteristics	Control group (n=47)	Test group (n=50)	*p*
Mean Age (± SD) at diagnosis (y)	44.11 (± 13.09)	40.76 (± 12.79)	0.206
Sex (%)			0.961
Female Male	34 (72.3%) 13 (27.6%)	37 (74.0%) 13 (26.0%)	
Cancer histology (%)			0.977
Papillary	46 (97.8%)	49 (98.0%)	
Follicular	1 (2.2%)	1 (2.0%)	
TNM classification (%)			
T1a	24 (51.1%)	25 (50%)	0.907
T1b	16 (34%)	16 (32%)	
T2	5 (10.6%)	6 (12%)	
T3	0	1 (2%)	
T4	0	0	
Unknown	2 (4.2%)	2 (4.0%)	
N0	2 (4.2%)	9 (18%)	0.060
N1a	27 (57.4%)	20 (40%)	
N1b	18 (38.3%)	21 (42%)	
M0	47 (100%)	50 (100%)	–
Disease stage (AJCC 8^th^)			0.223
I	37 (78.7%)	46 (92.0%)	
II	9 (19.1%)	4 (8.0%)	
Unknown	1 (2.1%)	–	
Risk of Recurrence (ATA 2025)			0.383
Low risk	3 (6.4%)	3 (6.0%)	
Low-intermediate/Intermediate-high risk	44 (93.6%)	45 (90.0%)	
Unknown	0 (0%)	2 (4.0%)	
TSH (± SD) (pre RAI)	80.45 (± 20.65)	77.47 (± 24.5)	0.601
sTG (± SD) (pre RAI)	2.04 (± 1.87)	1.56 (± 1.60)	0.337

RAI, radioactive iodine; sTg, stimulated thyroglobulin; TSH, Thyroid-stimulating hormone; SD, Standard Deviation.

### The rate of successful ablation

From structural perspective, rate of successful ablation was 93.3% in the control group and 91.2% in the test group (*p* = 0.748) for patients who underwent DxWBS 6–9 months after RAI therapy.

**Figure 2:**
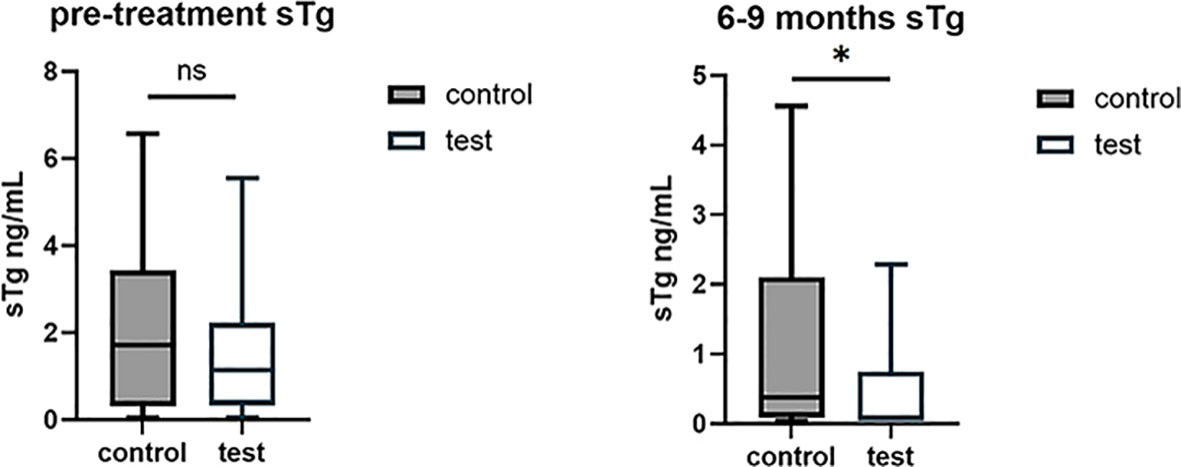
Difference of sTg between two groups. Serum sTg did not show any differences between the control and test group before RAI therapy **(A)**. Serum sTg was lower in the test group(RAI+ Lugol’s solution) than in the control group (RAI) at 6–9 months after RAI therapy **(B)**. sTg: stimulated thyroglobulin.

To evaluate successful ablation from biochemical level, sTg<1 ng/mL was used as the cutoff at the 6–9 months follow-up. It was demonstrated that addition of Lugol’s Solution administration led to numerically higher rate of successful ablation compared with test group (82.8% test vs. 66.7% control, *p* = 0.131). Further, we used less than 0.2 ng/mL as a stricter sTg cutoff to explore the efficacy between two groups, much higher successful ablation rate of test group was reported compared with control group (65.7% vs. 40.0%), showing statistical significance (*p* = 0.041). This suggested that more patients might achieve successful ablation with the addition of Lugol’s Solution. In addition, sTg was analyzed as continuous variable to evaluate effect of Lugol’s Solution administration on sTg. Results showed that Lugol’s Solution significantly decreased sTg level after RAI therapy compared with control group (0.09 vs. 0.38 ng/mL, *p* = 0.025) (**Figure 2**). Administration of Lugol’s Solution predicts decrease of sTg both in uni- or multi-variate analysis (**Table 2**).

**Table 2:** Univariable and multivariable linear regression model for stimulated Tg 6–9 months after radioactive iodine therapy.

	Uni-variate analysis			Multi-variate analysis		
Variable	Beta coefficient	SE	p	Beta coefficient	SE	p
Control group	Ref			Ref		
Test group	-0.559	0.232	**0.019**	-0.692	0.254	**0.009**
Female	Ref			Ref		
Male	0.273	0.267	0.311	0.372	0.283	0.194
Age	-0.001	0.01	0.948	-0.005	0.011	0.612
T stage						
T1a	Ref			Ref		
T1b	0.296	0.272	0.281	0.215	0.277	0.441
T2	0.065	0.367	0.861	0.084	0.367	0.820
T3	1.056	0.991	0.291	1.813	1.049	0.090
N stage						
N0	Ref			Ref		
N1a	0.075	0.390	0.940	-0.601	0.423	0.161
N1b	0.138	0.386	0.495	-0.191	0.417	0.648
Risk of Recurrence (ATA 2025)						
Low risk	Ref			Ref		
Low-intermediate/Intermediate-high risk	0.554	0.449	0.221	0.572	0.474	0.233
Unknown	-0.101	0.805	0.901	0.395	1.118	0.725
TSH	0	0.005	0.982	0.005	0.006	0.993

SE, Standard Error; sTg, stimulated thyroglobulin; Ref, reference.

Bold values indicate statistical significance (p < 0.05).

Apart from assessing structural response and biochemical response separately, we also combined no visible thyroid bed uptake and sTg less than 1 ng/mL as an indicator of successful ablation. The results demonstrated a higher rate with the addition of Lugol’s Solution, but it was not statistically significant (70.6% vs. 60.0%, *p* = 0.373).

### Adverse events

All AEs were Grade 1 by CTCAE v5.0. Toxicities profile on d3 are summarized in **Table 3**, showing 34% of patients in control group and 46% of patients in test group experienced adverse events without statistical significance (*p* = 0.230). The most common AEs included neck swelling and pain (12.7% vs. 22%, *p* = 0.231), loss of appetite (6.3% vs. 18%, *p* = 0.083) and dry mouth (8.5% vs. 20%, *p* = 0.101) in control and test group, respectively.

**Table 3:** Summary of adverse events (AEs).

	Control group (n=47)	Test group (n=50)	*p*
Total short-term AEs (d3)	16 (34.0%)	23 (46%)	0.230
Neck swelling and pain	6 (12.7%)	11 (22%)	0.231
Gastrointestinal disorders	11 (23.4)	18 (36%)	0.184
Loss of appetite	3 (6.3%)	9 (18%)	0.083
Dry mouth	4 (8.5%)	10 (20%)	0.101
Diarrhea	3 (6.4%)	2 (4%)	0.593
Nausea	1 (2.1%)	3 (6%)	0.336
Stomach bloating	0	2 (4%)	0.174
Constipation	2 (4.2%)	2 (4%)	0.948
Cough	1 (2.1%)	2 (4%)	0.591
Arthralgia	1 (2.1%)	0	0.301
Rash	1 (2.1%)	1 (2%)	0.967
Urinary tract pain	1 (2.1%)	0	0.301
Malaise	0	2 (4%)	0.174
Total short-term AEs (d10)	9 (19.1%)	15 (30%)	0.223
Neck swelling and pain	5 (10.6%)	7 (14%)	0.619
Gastrointestinal disorders	4 (8.5%)	11 (22%)	0.073
Loss of appetite	0	1 (2%)	0.330
Dry mouth	3 (6.3%)	10 (20%)	0.053
Diarrhea	1 (2.1%)	0	0.301
Nausea	0	1 (2%)	0.330
Stomach bloating	1 (2.1%)	1 (2%)	0.967
Cough	0	1 (2%)	0.330
Rash	1 (2.1%)	2 (4%)	0.591
Total long-term AEs (6–9 months)			
Dry eyes	1 (2.1%)	0	0.301
Dry mouth	1 (2.1%)	0	0.301

AEs, adverse events.

In terms of toxicities on d10 (**Table 3**), the incidence of all grade AEs decreased compared with toxicities on d3, suggesting gradual recovery of normal tissue. No new AEs were reported. The two groups still exhibited comparable AEs rates (19.1% vs.30%, *p* = 0.223). The most common AEs included neck swelling and pain (10.6% vs. 14%, *p* = 0.619) and dry mouth (6.3% vs. 20%, *p* = 0.053) in control and test group, respectively.

However, during 6- to 9-month follow-up period, all AEs in the test group were resolved, while one case of mild dry eye and one case of mild dry mouth persisted in the control group.

## Discussion

RAI therapy plays significant role in treatment of DTC (20). Numerous efforts have been made to optimize the clinical risk-benefit balance. In this preliminary study, the addition of Lugol’s Solution following RAI showed non-inferior ablation efficacy with a numerically lower incidence of long-term AEs, despite numerically higher short-term AE rates.

91.8% (89/97) patients were low-intermediate and intermediate-high risk group patients in this study, for whom RAI was may be considered both in ATA 2015 and in ATA 2025 guidelines (20, 21). By contrast, 6.2% (6/97) were low risk patients. The use of postoperative RAI ablation in low-risk group is still debated (22, 23). As per ATA 2025 guidelines, RAI is not recommended for low-risk patients. However, evidence is limited for patients with broader profiles, including those with N1a disease, or *BRAF* V600E–positive DTCs and young age group, in whom the biological behavior of thyroid cancer differs from that in older patients (24). As a result, if the low-risk patients are expected to have RAI, ATA 2025 guidelines also recommended dose of 30–50 mCi, reflecting clinical need for part of low-risk patient (21). Moreover, considering RAI may facilitate early detection of occult metastases, RAI for low-risk patients are recommended by Chinese Society of Nuclear Medicine, suggesting complexity of clinical practice (25). Taken together, the baseline characteristics of this study population reflects the evolution of clinical guidelines, real-world clinical practice, and patient preference. We believe that this preliminary study addresses a portion of clinical needs.

The Wolff-Chaikoff effect refers to the fact that after high iodine intake, the thyroid temporarily inhibits the organic process of iodine (i.e. the binding of iodine to thyroglobulin), thereby reducing the synthesis and release of thyroid hormone (T3/T4) (26). This may be explained by decreased TPO mRNA, impared I^-^ organification, leading to I^-^ accumulation in thyroid in the end (27). Consistent with this theory, the group with Lugol’s Solution demonstrated a numerically higher ablation success rate, with sTg<1 as cutoff (82.8% vs. 66.7%, *p* = 0.131), and a significantly higher rate, with sTg<0.2 as a stricter cutoff (65.7% vs. 40.0%, *p* = 0.041). Moreover, both univariate and multivariate analysis showed the administration of Lugol’s solution remained a significant independent predictor for decreased sTg levels.

Within the first 10 days of treatment, the test group (RAI+Lugol’s solution) exhibited more neck swell and pain, partly contributed to irradiation-induced cell death, inflammation, tissue swelling, etc. (28). This suggested that Lugol’s solution might increase iodine accumulation of the remnant thyroid and further enhance radioiodine ablation efficacy. However, with the normal tissue repairment, all neck swell and pain resolved in later phases (6–9 months after treatment). During early phase, incidence of dry mouth between two groups was similar (on d3, *p* = 0.101; on d10, *p* = 0.053). However, at 6–9 months after treatment, all patients in test group with dry mouth recovered, by contrast, one case in the control group did not recover from dry mouth. This may be partly attributed to the observation that higher iodide load in the circulation increases salivary iodide output (29), leading to reduced radiation-induced damage in salivary glands. In addition, a trend was observed that patients with Lugol’s Solution had more gastrointestinal toxicities, such as loss of appetite, nausea, etc. (30). According to our observation, this was more likely related to bitter and metallic taste of Lugol’s Solution. Therefore, it was recommended to take Lugol’s Solution together with juice or milk to mitigate gastrointestinal irritation (31). AEs were generally tolerable, clinically manageable and comparable between two groups. There was no AE leading to dose modification of Lugol’s solution. Quality of life (QoL) was not affected as reported by all patients. It was notably that assessment of QoL and quantification of normal tissue function (e.g., salivary glands) was not formally conducted in this study. To explore protective effect of Lugol’s Solution on normal tissues in the future, salivary gland function test and QoL questionnaires, such as European Organization for Research and Treatment of Cancer Quality of Life Questionnaire (EORTC QLQ-C30) and the Thyroid Cancer-Specific Quality of Life Questionnaire (THYCA-QOL), will be further conducted (32).

This study had several limitations. Firstly, patients with sTg> 10 ng/mL were excluded from this pilot study during study design phase with the concern that this population may not derive clinical benefit from Lugol’s Solution treatment. Exclusion of these patients may underestimate efficacy and safety difference between groups. Secondly, the use of lower-dose ^131^I (30mCi) resulted in a reduced incidence of AEs (33, 34), potentially obscuring the effect of the Lugol’s Solution in mitigating radiation-related toxicities. We speculated it may exhibit marked protective effect in later phase for patients receiving higher-dose RAI therapy, such as 100mCi or more, although it may enhance early-phase adverse effects due to increased iodine in remnant thyroid. Thirdly, quantification of normal tissue function, like salivary gland, after RAI could give us more insight into irradiation-induced damage, however, relative tests were not performed. Last but not least, due to the single-center design and small sample size of this preliminary clinical study, there may be limitations such as potential selection bias, a low incidence of AEs, and insufficient statistical significance in the results. Based on these data, to confirm the efficacy of Lugol’s Solution following RAI, multicenter clinical trial is needed. The study population will be broadened to include patients with pre-RAI sTg >10 ng/mL and those with additional high-risk profiles. Salivary function imaging, blood routine examination and other quantitative analysis of normal tissues function should be further performed to validate the safety of Lugol’s Solution following RAI therapy.

## Conclusion

In this preliminary study, the addition of Lugol’s Solution following RAI showed non-inferior ablation efficacy with a numerically lower incidence of long-term AEs, despite numerically higher short-term AE rates for the first time. Nevertheless, due to the small sample size and the low RAI activity that was administrated in the patients of our study, further studies with larger cohorts are needed to elucidate the potential beneficial role of Lugol’s solution as an add-on to RAI treatment.

## Data Availability

The raw data supporting the conclusions of this article will be made available by the authors, without undue reservation.
